# Complementary transcriptomic, lipidomic, and targeted functional genetic analyses in cultured *Drosophila *cells highlight the role of glycerophospholipid metabolism in Flock House virus RNA replication

**DOI:** 10.1186/1471-2164-11-183

**Published:** 2010-03-17

**Authors:** Kathryn M Castorena, Kenneth A Stapleford, David J Miller

**Affiliations:** 1 Departments of Internal Medicine, University of Michigan Medical School, Ann Arbor, MI 48109, USA; 2 Microbiology & Immunology, University of Michigan Medical School, Ann Arbor, MI 48109, USA; 3 Program in Cellular and Molecular Biology, University of Michigan Medical School, Ann Arbor, MI 48109, USA; 4 Section of Microbial Pathogenesis, Yale University School of Medicine, New Haven, CT 06536, USA

## Abstract

**Background:**

Cellular membranes are crucial host components utilized by positive-strand RNA viruses for replication of their genomes. Published studies have suggested that the synthesis and distribution of membrane lipids are particularly important for the assembly and function of positive-strand RNA virus replication complexes. However, the impact of specific lipid metabolism pathways in this process have not been well defined, nor have potential changes in lipid expression associated with positive-strand RNA virus replication been examined in detail.

**Results:**

In this study we used parallel and complementary global and targeted approaches to examine the impact of lipid metabolism on the replication of the well-studied model alphanodavirus Flock House virus (FHV). We found that FHV RNA replication in cultured *Drosophila *S2 cells stimulated the transcriptional upregulation of several lipid metabolism genes, and was also associated with increased phosphatidylcholine accumulation with preferential increases in lipid molecules with longer and unsaturated acyl chains. Furthermore, targeted RNA interference-mediated downregulation of candidate glycerophospholipid metabolism genes revealed a functional role of several genes in virus replication. In particular, we found that downregulation of *Cct1 *or *Cct2*, which encode essential enzymes for phosphatidylcholine biosynthesis, suppressed FHV RNA replication.

**Conclusion:**

These results indicate that glycerophospholipid metabolism, and in particular phosphatidylcholine biosynthesis, plays an important role in FHV RNA replication. Furthermore, they provide a framework in which to further explore the impact of specific steps in lipid metabolism on FHV replication, and potentially identify novel cellular targets for the development of drugs to inhibit positive-strand RNA viruses.

## Background

The relatively small genome of most positive-strand RNA viruses compels these pathogens to use cellular machinery to complete their replication cycles. The search for these "host factors" utilized by positive-strand RNA viruses is at the forefront of virology research, due in part to the possibility that cellular proteins or processes may represent more stable drug targets or provide broader antiviral activity when disrupted [1]. One diverse host factor that has been identified as crucial for positive-strand RNA virus replication are intracellular membranes [2-5]. Although viruses that contain a lipid envelope as a structural component clearly utilize cellular membranes to form infectious virions, all positive-strand RNA viruses, both enveloped and non-enveloped, also depend on host intracellular membranes for the assembly and function of the viral RNA replication complexes essential for genome amplification. The precise functions of cellular membranes in this process have not been fully defined, but may include: (i) serving as structural scaffolds for replication complex targeting and assembly; (ii) protecting viral RNA or replication intermediates from cellular antiviral defense responses; or (iii) providing essential protein or lipid cofactors for optimal viral enzymatic activities. These proposed functions are not mutually exclusive, and it is likely that cellular membranes and their constituent components play multiple roles in viral RNA replication.

**Figure 1:**
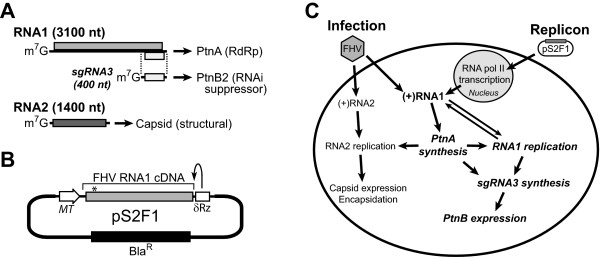
**FHV genome and replicon schematics and replication strategy**. (A) FHV genome is bipartite with 3.1 kb (RNA1) and 1.4 kb (RNA2) segments, and during replication a 0.4 kb subgenomic (sg) segment (RNA3) is also produced. RNA1 encodes protein A, the FHV RNA-dependent RNA polymerase (RdRp), RNA2 encodes the FHV structural protein precursor, and RNA3 encodes the RNA interference (RNAi) suppressor protein B2 (PtnB2). (B) Schematic of pS2F1, a metallothionein (MT) promoter-driven plasmid that expresses an FHV RNA1 replicon. Authentic viral 5' and 3' termini are generated by precise transcription initiation and a hepatitis δ ribozyme (δRz), respectively. The asterisk indicates the position of the frame-shifting mutation in pS2F1_fs _that results in the production of a truncated and non-functional RNA polymerase. Expression of the blasticidin-resistance gene (Bla^R^) used for selection is controlled by the *copia *transposon LTR constitutive promoter. (C) Strategy for FHV genome replication initiated by infection or replicon transfection. The intracellular events indicated in bold type are common for FHV RNA replication initiated via either infection or replicon induction.

To investigate the role of host factors in viral RNA replication we use *Flock House virus *(FHV), a versatile model virus and natural insect pathogen that assembles robust functional RNA replication complexes in yeast [6,7], plant [8], mammalian [9], nematode [10], and insect cells [11]. This broad array of eukaryotic hosts that support FHV RNA replication suggests that cellular factors utilized by this virus are widely conserved. The FHV genome is bipartite, with two positive-sense RNA segments copackaged into a non-enveloped virion (Fig. 1A). The larger 3.1-kb genomic segment, RNA1, encodes protein A, the FHV RNA-dependent RNA polymerase, which is the only viral protein required for functional RNA replication complex assembly. FHV assembles its RNA replication complexes on mitochondrial outer membranes [7,11], where they are targeted and anchored in part via an amino-proximal transmembrane domain present within protein A [12]. During RNA replication FHV produces a 0.4-kb subgenomic segment, RNA3, which encodes the RNA interference (RNAi) suppressor protein B2. This viral counterdefense protein is required for maximal viral RNA synthesis in cells with an active RNAi antiviral system [10,13]. Since subgenomic RNA3, and hence protein B2, are produced only during active RNA replication, they serve as convenient and quantitative markers for FHV RNA replication complex activity. The smaller 1.4-kb genomic segment, RNA2, encodes the structural capsid protein precursor. The FHV capsid protein is required for infectious virion production but is dispensable for viral RNA replication, and therefore engineered self-replicating viral RNAs, termed replicons, need only contain FHV RNA1 (Figs. 1B and 1C).

Apart from the membrane localization of its viral RNA replication complexes, additional lines of evidence point to the importance of membranes, and in particular lipids, in FHV replication. Protein A is a lipid-binding protein with particular affinity for specific anionic phospholipids [14], which may mediate in part the protein A-membrane interactions required for replication complex assembly. FHV RNA replication induces dramatic mitochondrial membrane rearrangements [7,11,15,16], reminiscent of structures seen with other positive-strand RNA viruses [2]. The fatty acid synthetase inhibitor cerulenin disrupts FHV RNA replication [17], consistent with the activity of this inhibitor on other positive-strand RNA viruses [18,19]. Finally, FHV RNA replication complex activity in isolated membrane fractions analyzed in vitro is disrupted by certain detergents [20], and can be augmented by the addition of exogenous phospholipids [21]. These results all suggest a central role of cellular lipid metabolism in FHV RNA replication complex assembly and function. However, none of them provide direct evidence for a functional impact of specific lipid metabolism pathways on FHV RNA replication within intact cells.

In this report, we use complementary transcriptomic, lipidomic, and targeted functional genetic analyses to specifically examine the role of lipids in FHV RNA replication in cultured *Drosophila *cells. We demonstrate that FHV infection or replicon expression upregulates the transcription of a distinct set of cellular genes, several of which are involved in lipid metabolism. Furthermore, FHV RNA replication induces global changes in cellular phospholipid content, and in particular phosphatidylcholine (PC), and pharmacologic or genetic disruption of PC synthesis within cells inhibits FHV RNA replication.

## Results

### FHV RNA replication induces transcriptional upregulation of lipid metabolism genes in *Drosophila *S2 cells

We examined the transcriptional responses of *Drosophila *S2 cells to FHV RNA replication using genome-wide microarray analyses. To focus on cellular responses related to RNA replication rather than those associated with other steps in the viral life cycle, such as virion attachment, entry, uncoating, encapsidation, and virion release, we conducted parallel analyses of cells either infected with FHV or transfected with an RNA1 replicon-expression plasmid. The RNA1 segment of the FHV genome encodes the viral RNA-dependent RNA polymerase (Fig. 1A) and is the only genome segment required for RNA replication (Fig. 1C). Thus, FHV RNA replication can be initiated in S2 cells by the introduction of a self-replicating RNA1 via the inducible plasmid pS2F1 (Figs. 1B and 1C) [22]. To maximize the number of S2 cells expressing the replicon, we first generated stable cell lines containing pS2F1 or a control plasmid (pS2F1_fs_, see Fig. 1B legend) containing a translation-defective RNA1 segment. Although there was a low level of baseline FHV RNA replication in pS2F1-expressing cells due to leaky metallothionein promoter activity, induction with copper sulfate resulted in a dramatic increase in subgenomic RNA3 and protein B2 accumulation, indicative of highly active viral RNA replication complexes (data not shown).

**Figure 2:**
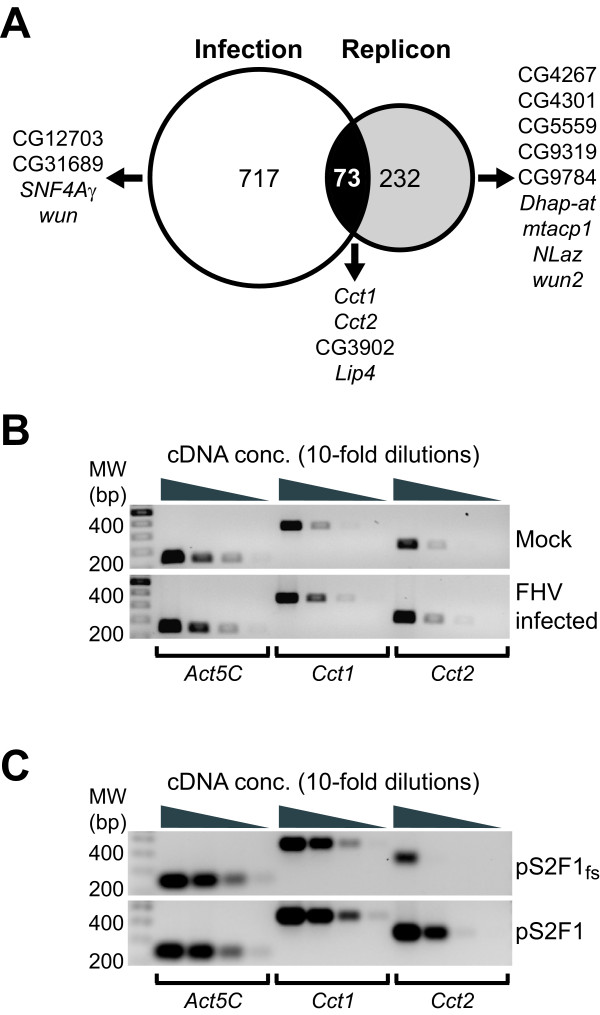
**FHV infection and replicon expression upregulate partially overlapping sets of *Drosophila *genes**. (A) Venn diagram indicating the number of upregulated genes unique to FHV-infected cells (white circle), unique to FHV replicon-expressing cells (grey circle), or upregulated with both (black convergence). Total numbers of genes are given within the indicated regions, and complete lists and descriptions of genes are provided in Table 1 and as Additional Files 1, 2 and 3. Specific upregulated genes involved in lipid metabolism, as identified by GO terms, are shown by either their gene symbols or CG designations. (B) Semi-quantitative RT-PCR validation of *Cct1 *and *Cct2 *mRNA upregulation in *Drosophila *S2 cells infected with FHV. Decreasing amounts of cDNA generated by RT with oligo-dT primers and equivalent amounts of total RNA from mock (upper gel) or FHV-infected S2 cells (lower gel) were amplified with gene-specific primers for *Drosophila *actin (*Act5C)*, *Cct1*, or *Cct2*, and PCR products were examined by agarose gel electrophoresis and ethidium bromide staining. The expression level of the *Act5C *transcript in microarray experiments was not significantly altered with FHV infection or replicon expression. Densitometry analysis of PCR products generated from cDNA dilutions that produced submaximal signals showed that FHV infection induced 2.0 ± 0.2 and 2.3 ± 0.3 fold increases in *Cct1 *and *Cct2 *mRNA levels, respectively, consistent with quantitative microarray results (see Additional File 1). (C) Semi-quantitative RT-PCR validation of *Cct1 *and *Cct2 *mRNA upregulation in *Drosophila *S2 cells expressing an FHV replicon. RT-PCR was performed as described above with total RNA from S2 cells containing the control plasmid pS2F1_fs _(upper gel) or FHV replicon-encoding plasmid pS2F1 (lower gel). Densitometry analysis of PCR products as described above showed that FHV replicon expression induced 1.9 ± 0.2 and 2.8 ± 0.9 fold increases in *Cct1 *and *Cct2 *mRNA levels, respectively, consistent with quantitative microarray results (see Additional File 2).

We analyzed transcript levels from three independent experiments with either FHV-infected cells or cells expressing an RNA1 replicon, and identified cellular genes whose transcription was significantly up- or downregulated in response to these stimuli. We identified 790 genes that were significantly upregulated and 392 genes that were significantly downregulated in FHV-infected S2 cells (Additional File 1), and 305 genes that were significantly upregulated in pS2F1-expressing cells (Additional File 2). There were no significantly downregulated genes in pS2F1-expressing cells. Full details regarding the molecular functions, biological processes, and cellular components for these genes are provided in the additional files. We further analyzed these datasets for transcripts that were upregulated with both stimuli and identified 73 co-regulated genes, which encoded proteins involved in numerous aspects of cellular physiology, such as metabolism, defense responses, and signal transduction (Table 1 and Additional File 3). Based in part on the well recognized importance of cellular membranes in positive-strand RNA virus replication [3-5], we focused on those genes that encoded proteins with known or hypothesized roles in lipid metabolism. We found that infection with FHV upregulated eight lipid metabolism-associated genes, whereas RNA1 replicon expression upregulated thirteen lipid metabolism-associated genes (Fig. 2A). Four of these genes were upregulated in both datasets, including *Cct1 *and *Cct2*, which encode CTP:phosphocholine cytidylyltransferases. These enzymes are essential for the salvage pathway of PC synthesis but have non-overlapping functions in *Drosophila *cells [23-26]. We validated both *Cct1 *and *Cct2 *mRNA upregulation by RT-PCR after FHV infection (Fig. 2B) and RNA1 replicon expression (Fig. 2C). These results demonstrated that FHV RNA replication in *Drosophila *S2 cells was associated with distinct alterations in cellular gene transcription, including the transcription of genes associated with lipid metabolism.

**Table 1:** *Drosophila *genes co-upregulated in S2 cells either infected with FHV or expressing an RNA1 replicon.

Biological Process	Gene Symbol	Gene Name
Carbohydrate metabolism	cenB1A	Centaurin β1A
	
	CG12582	-

Heme metabolism	Alas	Aminolevulinate synthase
	
	l(3)02640	Lethal (3) 02649

Lipid metabolism	Cct1	CTP:phosphocholine cytidylyltransferase 1
	
	Cct2	CTP:phosphocholine cytidylyltransferase 2
	
	CG3902	-
	
	Lip4	Lipase 4

Protein metabolism	CalpC	Calpain C
	
	CG1340	-
	
	CG4266	-
	
	CG18557	-
	
	dream	dream
	
	pUf68	Poly U binding factor 68 kDa
	
	stv	Starvin

Metabolism, other	CG3168	-
	
	CG9886	-
	
	CG10137	-
	
	CG17807	-

Defense response	cactin	cactin
	
	dos	Daughter of sevenless
	Eip75B	Ecdysone-induced protein 75B
	
	PGRP-LA	Peptidoglycan recognition protein LA
	
	Rel	Relish
	
	Sp7	Serine protease 7
	
	Traf-like	TNF-receptor-associated factor-like

Signal transduction	CG6954	-
	
	CG11534	-
	
	CG12091	-
	
	Dgp-1	Dgp-1
	
	drongo	drongo
	
	Ptp61f	Protein tyrosine phosphatase 61F
	
	RhoGAP18B	RhoGAP18B
	
	Socs36E	Suppressor of cytokine signalling at 36E
	
	sra	Sarah

Transcription	CG16903	-
	
	Ets21C	Ets at 21C
	
	Nvy	Nervy
	
	Rab10	Rab-protein 10

Cytoskeletal organization	Insc	Inscuteable
	
	WASp	WASp

Transport	CG4726	-
	
	Tamo	tamo

DNA synthesis	DNApol-iota	DNApol-iota
	
Stress response	Hsp23	Heat shock protein 23
	
Cell development	Foi	Fear-of-intimacy
	
Ubiquitin cycle	CG9153	-
	
Unknown	CG1529	-
	
	CG4036	-
	
	CG4281	-
	
	CG5118	-
	
	CG5399	-
	
	CG6701	-
	
	CG6762	-
	
	CG7326	-
	
	CG7457	-
	
	CG7967	-
	
	CG8620	-
	
	CG9796	-
	
	CG12022	-
	
	CG12084	-
	
	CG13117	-
	
	CG13917	-
	
	CG13926	-
	
	CG14642	-
	
	CG33191	-
	
	CG34349	-
	
	CG42324	
	
	Cyp6a17	Cyp6a17
	
	meso18E	meso18E
	
	Msr-110	Msr-110
	
	unc-119	unc-119

Biological process designation was determined from either direct GO biological process terms or inferred from GO molecular function terms curated from the Flybase database http://flybase.org/. Complete data for co-upregulated genes, including Flybase ID, CCG number, gene symbol, gene name, GO terms (function, process, and compartment), genetic interaction partners, yeast and human orthologs, and fold change are provided as Additional File 3.

### FHV RNA replication alters phospholipid levels in *Drosophila *S2 cells

**Figure 3:**
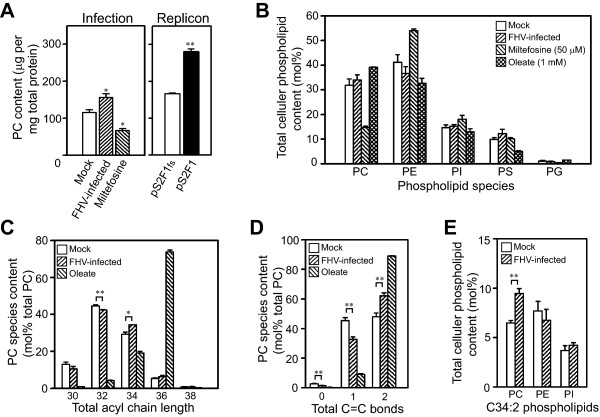
**FHV infection and replicon expression modulate phospholipid levels in *Drosophila *S2 cells**. (A) Total PC levels in S2 cells infected with FHV or treated with miltefosine (left graph) or expressing an FHV RNA1 replicon (right graph). Total PC levels were determined in cells 24 h after infection, treatment, or replicon induction and are expressed relative to total cellular protein levels. (B) Phospholipid species distribution in control S2 cells, cells infected with FHV, or cells treated with miltefosine or oleic acid. Relative phospholipid species content is expressed as a molar percentage of total phospholipid content and was determined by ESI-MS/MS. (C) PC acyl chain length in mock, FHV-infected, and oleic acid-treated S2 cells. Total PC chain length was determined by ESI-MS/MS and represents the total number of carbons from both acyl chains. (D) PC acyl chain saturation in mock, FHV-infected, and oleic acid-treated S2 cells. Total number of C = C double bonds in PC species was determined by ESI-MS/MS and represents the total number of double bonds in both acyl chains. The fraction of PC species with greater than two double bonds was 0.5 to 1.5% for both mock and FHV-infected cells (see Additional File 4), and therefore these results were not included in the graph. (E) PC, PE, and PI species with acyl chains containing a total of 34 carbons and 2 double bonds in mock and FHV-infected S2 cells. *P*-value < 0.05* or 0.005**.

The conclusion that FHV RNA replication was associated with transcriptional upregulation of lipid metabolism genes predicts that quantifiable changes in lipid constituents should also be observed, particularly with respect to PC content. To examine this prediction we measured total PC levels in S2 cells after infection or replicon expression using a modified phospholipase D-based biochemical assay [27] (Fig. 3A). As a control for these experiments we used cells treated with 1-hexadecylphosphorylcholine (miltefosine), which is a specific CTP:phosphocholine cytidylyltransferase inhibitor [28]. S2 cells infected with FHV had a 35% increase in total PC levels, whereas treatment with miltefosine reduced PC levels by 40% (Fig. 3A, left graph). Furthermore, although baseline PC levels in stably transfected cells were increased compared to untransfected cells, expression of an FHV RNA1 replicon was associated with a 70% increase in PC levels (Fig. 3A, right graph). These results indicated that FHV RNA replication-associated transcriptional upregulation of PC biosynthetic genes in S2 cells (Fig. 2) was associated with a concomitant increase in cellular PC levels.

To evaluate potential lipid changes associated with FHV replication in S2 cells in more detail we used electrospray ionization tandem mass spectrometry (ESI-MS/MS) to quantitate polar phospholipids in extracts from FHV-infected cells. This analysis allowed the determination of relative individual phospholipid species content as a molar percentage of total recovered polar phospholipids but also provided details on total acyl chain length and saturation levels. As controls for these experiments we used cells treated with miltefosine to decrease PC levels or cells cultured with 1 mM oleic acid (*cis*-9-octadecenoic acid), which increases the production of phospholipids with individual C18:1 acyl chains and hence total sum acyl chain compositions of C36:2. Complete detailed results from the ESI-MS/MS analyses are provided in Additional File 4. Control membrane extracts from S2 cells contained predominantly PC and phosphatidylethanolamine (PE) as the primary phospholipids, with the latter representing the majority (~40%) of all phospholipids (Fig. 3B). Although PC is typically the most prominent phospholipid in most eukaryotic cell membranes, *Diptera *species such as *Drosophila *contain relatively high levels of PE [29]. As expected, miltefosine dramatically reduced PC content with compensatory increases in the percentages of PE and phosphatidylinositol (PI). In contrast, total cellular membranes from FHV-infected S2 cells showed a trend towards an increase in the molar percentage of PC and phosphatidylserine (PS) with an associated decrease in PE content, but these differences were not statistically significant (Fig. 3B). However, when we analyzed PC species based on total acyl chain length or saturation level there were modest but statistically significant changes in the percentage of PC molecules with total acyl chain lengths of 32 or 34 carbons (Fig. 3C) and 0, 1, or 2 total double bonds (Fig. 3D), where FHV infection increased the fraction of PC molecules with longer unsaturated acyl chains. Furthermore, the selective increase in longer unsaturated acyl chains in FHV-infected cells was seen specifically with PC and not PE, PI, or PS (Fig. 3E and data not shown). These quantitative lipid analysis results suggested that FHV infection induces selective changes in PC metabolism, and are consistent with the observation that FHV RNA replication complex activity in isolated membrane fractions analyzed in vitro is preferentially stimulated by phospholipids with increased acyl chain length and decreased saturation [21].

### Manipulation of glycerophospholipid metabolism gene expression modulates FHV RNA replication in *Drosophila *S2 cells

**Figure 4:**
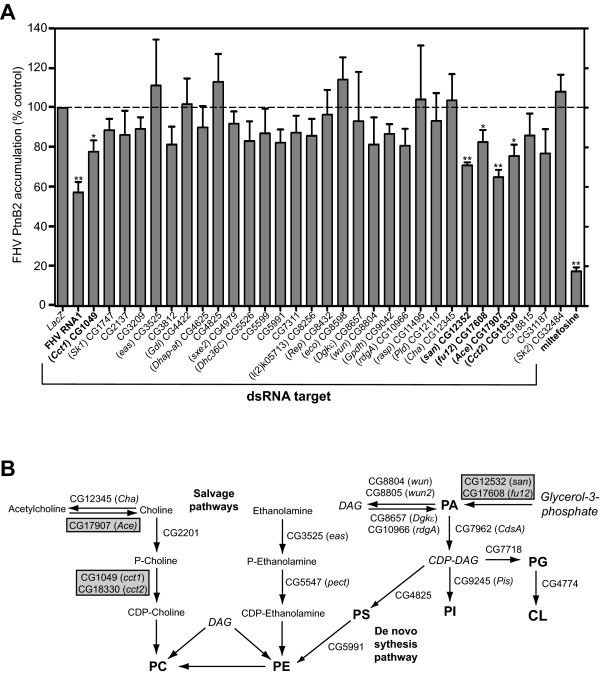
**RNAi-mediated knockdown of *Drosophila *genes involved in glycerophospholipid metabolism modulate FHV replication in S2 cells**. (A) Medium throughput RNAi-based functional screen of select glycerophospholipid metabolism genes. *Drosophila *S2 cells were cultured in 96-well plates, incubated with dsRNA targeting specific glycerophospholipid metabolism-related genes, positive control FHV RNA1, or negative control *LacZ *for 48 h, infected with FHV, and harvested 18 h after infection. As an additional separate control, selected wells were treated with 50 μM miltefosine at the time of infection. FHV replication was assayed by protein B2 (PtnB2)-specific capture ELISA. Parallel MTT viability assays demonstrated > 90% viability for all dsRNA- or miltefosine-treated samples compared to *LacZ *dsRNA-treated control (data not shown). Results are expressed as the percentage of PtnB2 accumulation relative to *LacZ *dsRNA-treated control (hatched line). *P*-value < 0.05* or 0.005**. (B) Schematic of eukaryotic glycerophospholipid synthesis pathways. The simplified biosynthetic pathways shown were adapted from the detailed and comprehensive KEGG glycerophospholipid metabolism pathway available at http://www.genome.jp/kegg/. *Drosophila *genes with known or hypothesized lipid biosynthetic functions are shown, and those identified as being functionally important for FHV RNA replication are boxed in grey. The major eukaryotic glycerophospholipids are indicated in bold type: CL, cardiolipin; PA, phosphatidic acid; PC, phosphatidylcholine; PE, phosphatidylethanolamine; PG, phosphatidylglycerol; PI, phosphatidylinositol; PS, phosphatidylserine. The essential precursor glycerol-3-phosphate and important intermediates are shown in italics: DAG, diacylglycerol.

We next examined the potential functional significance of lipid metabolism gene upregulation on FHV RNA replication in S2 cells using a targeted genetic approach that employed RNAi-mediated knockdown [17]. We identified thirty-one cDNAs within the O'Farrell RNAi collection [30] that were targeted toward genes involved in glycerophospholipid metabolism in *Drosophila *cells, generated 300-600 bp dsRNAs, and performed RNAi knockdown experiments with infected S2 cells using dsRNA targeted against FHV RNA1 and miltefosine as positive controls (Fig. 4A). To quantitate FHV RNA replication, we used an optimized capture enzyme-linked immunosorbant assay (ELISA) that detected protein B2, which is a viral protein only produced during active RNA replication (see Fig. 1C). Although protein B2 is an RNAi suppressor, it functions during the assembly of RNAi-induced silencing complexes [31], and therefore would have less impact on the activity of functional silencing complexes formed during the 48 h dsRNA incubation period prior to FHV infection. We found that dsRNAs directed against five cellular genes, *Ace*, *Cct1*, *Cct2*, *fu12*, and *san*, all significantly suppressed FHV RNA replication in infected S2 cells (Fig. 4A). Although the level of suppression was 20-40% with individual knockdown of these five genes, the positive control FHV dsRNA only reduced protein B2 levels by ~45%, suggesting that this medium throughput assay provided a conservative estimate of the functional impact of cellular glycerophospholipid metabolism gene knockdown. The functions of *Cct1 *and *Cct2 *have been described above. *Ace *encodes acetylcholine esterase [32], *fu12 *encodes an enzyme with 1-acylglycerol-3-phosphate acyltransferase activity [33], and *san *also encodes an enzyme with acyltransferase activity [34]. The enzymes encoded by *Cct1*, *Cct2*, *Ace*, *fu12*, and *san *are all involved in PC synthesis, either directly or indirectly (Fig. 4B). The main glycerophospholipids in the cell, PC and PE, are synthesized through two metabolic pathways, the de novo synthesis pathway that uses PS as a precursor for both, and the salvage or CDP pathway, also referred to as the Kennedy pathway, which recycles choline and ethanolamine to produce PC and PE, respectively [35]. In higher eukaryotes the salvage or CDP pathway of PC synthesis normally predominates, where the conversion of phosphocholine to CDP-choline, which is catalyzed by CTP:phosphocholine cytidylyltransferases such as those encoded by *Cct1 *and *Cct2*, is the rate limiting step [35]. Thus, we chose to focus further on these two *Drosophila *glycerophospholipid synthesis genes.

**Figure 5:**
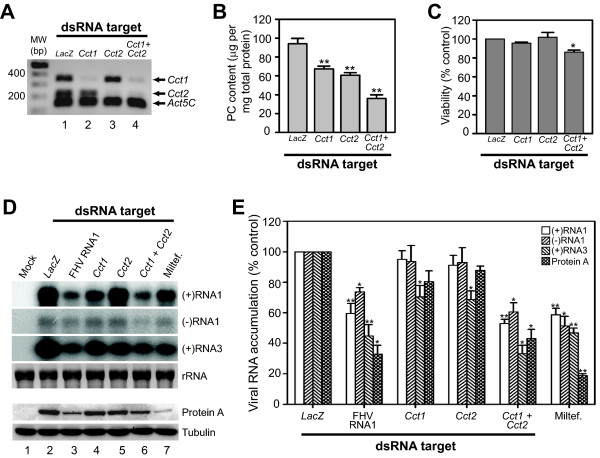
**Verification of *Cct1 *and *Cct2 *roles in modulating FHV replication in infected S2 cells**. (A) RT-PCR validation of *Cct1 *or *Cct2 *RNAi-mediated knockdown. *Drosophila *S2 cells were treated with dsRNAs specific for *LacZ *(lane 1), *Cct1 *(lane 2), *Cct2 *(lane 3), or both *Cct1 *and *Cct2 *(lane 4) for 72 h, and gene-specific mRNA expression was examined by semi-quantitative RT-PCR as described in Fig. 2, except that only results from cDNA dilutions that produced submaximal signals are shown. (B) Total PC content in cells treated with the dsRNAs described above. PC levels were determined as in Fig. 3. (C) Viability of cells treated with dsRNAs described above determined by MTT assay. (D) FHV RNA accumulation in infected S2 cells after RNAi-mediated knockdown of *Cct1*, *Cct2*, or both. Mock-infected cells (lane 1), cells treated with the indicated dsRNA as described above or FHV RNA1 as a positive control (lanes 2-6), or treated with 50 μM miltefosine (lane 7), were infected with FHV and viral-specific RNAs or protein were analyzed by northern blotting or immunoblotting 12 h after infection, respectively. For viral RNA analysis, blots for positive-sense (+) and negative-sense (-) genomic RNA1 and subgenomic (+)RNA3 are shown. The decrease in (+)RNA1 accumulation in *Cct1 *knockdown cells (lane 4) was not consistently seen in all experiments. Ribosomal RNA (rRNA) bands detected by ethidium bromide staining are shown as loading controls. For protein analysis, blots for FHV protein A and the cellular loading control tubulin are shown. (E) Quantitative data for genomic (+)RNA1 and (-)RNA1, subgenomic (+)RNA3, and protein A accumulation in S2 cells treated with the indicated dsRNA or miltefosine after infection with FHV. Results are expressed as percentage of accumulation relative to *LacZ *dsRNA-treated control. *P*-value < 0.05* or 0.005**.

To verify the functional impact of *Cct1 *and *Cct2 *activity on FHV RNA replication in S2 cells, we generated alternative dsRNAs targeting different coding regions of these two genes and repeated RNAi-mediated knockdown experiments, including co-knockdown of both *Cct1 *and *Cct2 *mRNA (Fig. 5). We validated knockdown of *Cct1 *and *Cct2 *mRNA expression by RT-PCR, where we obtained an approximate 80-90% gene-specific reduction in steady state levels (Fig. 5A). Furthermore, quantitative analysis demonstrated a 30-35% reduction in total PC levels with individual knockdown of either *Cct1 *or *Cct2 *mRNA, and a ~60% reduction with knockdown of both genes (Fig. 5B). The incomplete knockdown of *Cct1 *and *Cct2 *mRNA, the presence of a *Cct*-independent de novo PC synthesis pathway (see Fig. 4B), and the essential role that PC plays in cellular structure and metabolism all likely contributed to the lack of synergistic suppression of total PC levels with an RNAi-based approach that targeted one step in the biosynthetic pathway. We also examined cell viability by MTT assay, as disruption of CTP:phosphocholine cytidylyltransferase expression or function in mammalian cells results in enhanced susceptibility to apoptosis [36-38]. Individual knockdown of either *Cct1 *or *Cct2 *mRNA had no significant impact on cell viability, whereas co-knockdown of both genes resulted in a small but reproducible 15% reduction in cell viability as measured by MTT assay (Fig. 5C). We infected S2 cells with FHV after RNAi-mediated suppression of *Cct1*, *Cct2*, or both genes, and measured viral RNA and protein accumulation at 12 h after infection. Downregulation of either *Cct1 *or *Cct2 *individually suppressed FHV subgenomic (+)RNA3 accumulation by ~30%, but did not significantly reduce the accumulation of genomic (+)RNA1, (-)RNA1, or protein A (Figs. 5D and 5E). However, downregulation of both *Cct1 *and *Cct2 *resulted in a 40-65% reduction in the accumulation of all viral products examined, similar to the levels of suppression seen with the positive controls miltefosine and dsRNA directly targeting FHV RNA1. Although we cannot exclude the possibility that the small reduction in cell viability with co-knockdown of both *Cct1 *and *Cct2 *(Fig. 5C) adversely affected viral RNA or protein accumulation, potentially via apoptosis-mediated reduction in synthesis or stability, published studies have demonstrated that FHV replication is not sensitive to the antiviral effects of apoptosis induction in *Drosophila *cells [39]. These results indicated that genetic or pharmacologic disruption of PC synthesis inhibited FHV RNA replication in infected S2 cells.

**Figure 6:**
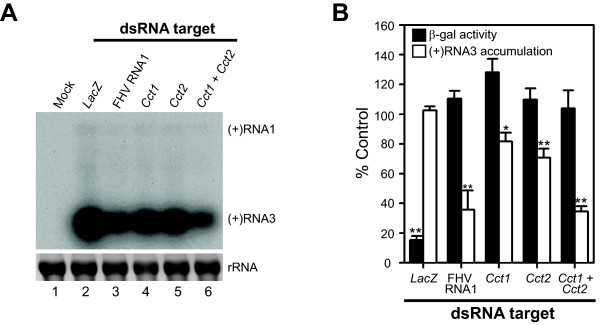
**Verification of *Cct1 *and *Cct2 *roles in modulating FHV RNA replication in replicon-bearing S2 cells**. (A) Northern blot of FHV genomic (+)RNA1 and subgenomic (+)RNA3 accumulation in FHV replicon-bearing S2 cells after RNAi-mediated knockdown of *Cct1*, *Cct2*, or both. Mock transfected cells (lane 1) or cells treated with dsRNA against *LacZ *(lane 2), FHV RNA1 (lanes 3), *Cct1 *(lane 4), *Cct2 *(lane 5), or both *Cct1 *and *Cct2 *(lane 5) and co-transfected with pS2F1 and pS2LacZ were induced with 0.5 mM Cu^2+ ^for 18 h and FHV RNA accumulation was determined by northern blotting as described in Fig. 5. The position of FHV subgenomic (+)RNA3 is shown on the right, and ribosomal RNA (rRNA) bands are shown as loading controls. The position of genomic (+)RNA1, which is barely detectable in transiently transfected S2 cells, is also shown on the right. (B) Quantitative data for subgenomic (+)RNA3 and β-galactosidase activity in S2 cells co-transfected with pS2F1 and pS2LacZ after RNAi-mediated knockdown of the indicated dsRNA targets. *P*-value < 0.05* or 0.005**.

To further examine the functional impact of *Cct1 *and *Cct2 *on FHV RNA replication in the absence of infectious virus, we repeated experiments using RNAi-mediated downregulation of these genes in S2 cells expressing an RNA1 replicon (Fig. 6). We could not use stably transfected cells for genetic knockdown experiments, as the low level of FHV RNA replication in these cells in the absence of copper sulfate induction resulted in protein B2 production that inhibited RNAi-mediated downregulation of cellular genes (data not shown). Therefore, we used a transient transfection approach. We treated S2 cells with dsRNAs for 48 h, transfected cells with pS2F1 (see Fig. 1B) and control plasmid pS2LacZ, and measured FHV RNA replication by northern blotting and β-galactosidase activity by enzymatic assay 18 h after induction. We used pS2LacZ as a transfection control, as modulation of PC levels may influence transfection efficiency. Knockdown of either *Cct1 *or *Cct2 *suppressed FHV (+)RNA3 accumulation by 20-40% in pS2F1-transfected cells, whereas knockdown of both genes increased suppression to 65%, similar to levels seen with control dsRNA targeted against FHV RNA1 (Figs. 6A and 6B). We did not quantitate genomic (+)RNA1 or protein A accumulation levels in transfected cells, as these viral products accumulated to low and almost undetectable levels in pS2F1-transfected cells (Fig. 6A and data not shown), such that attempts at quantitation would have been unreliable. Nevertheless, these results supported the conclusion that disruption of PC synthesis by genetic modulation of *Cct1 *or *Cct2 *inhibited FHV RNA replication.

**Figure 7:**
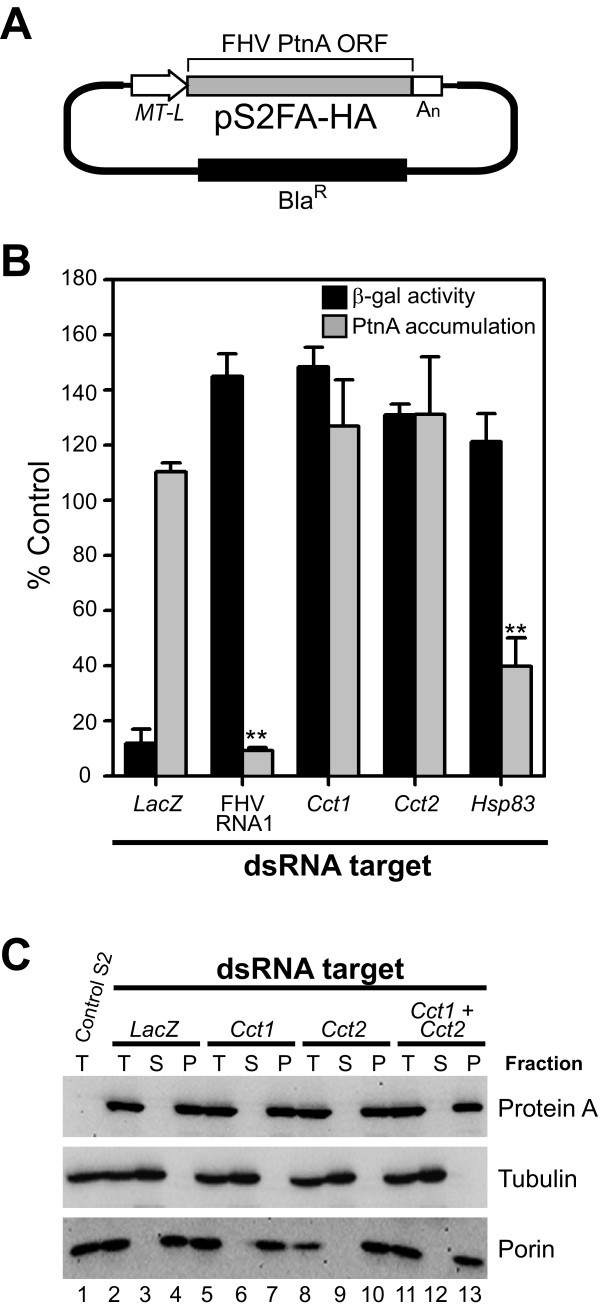
**RNAi-mediated knockdown of *Cct1 *or *Cct2 *does not modulate FHV protein A accumulation or membrane association in S2 cells**. (A) Schematic of FHV protein A expression vector with C-terminal HA tag. The RNA template produced from pS2FA-HA is optimized for translation by inclusion of a 5' leader sequence (L) and a 3' polyadenylation signal (A_n_), but these alterations in addition to the C-terminal HA tag render the RNA template incompetent for viral RNA replication due to changes in essential 5' and 3' cis elements. (B) FHV protein A accumulation determined by quantitative immunoblotting and β-galactosidase activity determined by enzyme assay in S2 cells co-transfected with pS2FA-HA and pS2LacZ after RNAi-mediated knockdown of the indicated dsRNA targets. *P*-value < 0.05* or 0.005**. (C) S2 cells stably expressing either an empty vector (lane 1) or pS2FA-HA (lanes 2-13) were treated with dsRNA targeting *LacZ *(lanes 2-4), *Cct1 *(lanes 5-7), *Cct2 *(lanes 8-10), or both *Cct1 *and *Cct2 *(lanes 11-13) for 72 h, induced with 0.5 mM Cu^2+ ^for 18 h, washed in PBS, and either lysed directly in SDS-PAGE buffer to obtain total fractions (T) or subjected to saponin-mediated permeabilization and differential centrifugation to obtain soluble (S) and pellet (P) fractions, which correspond to cytosolic and membrane protein fractions, respectively [22]. Fractions were analyzed by SDS-PAGE and immunoblotting for protein A, the cytosolic protein tubulin, or the membrane protein porin.

Finally, we examined whether disruption of *Cct1 *or *Cct2 *expression directly suppressed FHV protein A accumulation or membrane association (Fig. 7), as we have previously demonstrated that cellular factors can impact viral RNA polymerase production [17,22] and hence facilitate an early step in RNA replication complex assembly (see Fig. 1C). Although protein A levels were decreased in FHV-infected cells with RNAi-mediated downregulation of both genes (Figs. 5D and 5E), protein A accumulation in the setting of infectious virus or an FHV replicon is directly linked to RNA replication (see Fig. 1C). Thus, we used a protein A expression vector, called pS2FA-HA (Fig. 7A), which is designed to optimize translation and prevent RNA replication via modification of 5' and 3' untranslated sequences that contain essential cis elements [17,22]. In contrast to the effects of *Cct1*- or *Cct2*-specific dsRNAs on FHV RNA replication (Figs. 5 and 6), knockdown of either gene had no effect on RNA polymerase accumulation in cells transfected with pS2FA-HA (Fig. 7B), whereas dsRNAs against *Hsp83 *suppressed polymerase accumulation by 60%, consistent with published studies demonstrating the important role of hsp90 chaperones in FHV protein A synthesis [17,22]. Furthermore, knockdown of *Cct1*, *Cct2*, or both genes did not alter the ability of protein A to associate with intracellular membranes, as determined by differential centrifugation (Fig. 7C). These results indicated that PC synthesis was important for FHV RNA replication but not viral polymerase production or membrane association.

## Discussion

Global approaches such as transcriptomic, proteomic, and functional genomic analyses have provided important clues to critical host-pathogen interactions that influence virus replication and pathogenesis [40-47]. However, these approaches when used in isolation often provide an overwhelming amount of information that requires careful selection and validation. We have used an alternative approach that incorporates more targeted analyses including lipidomics to specifically examine the role of glycerophospholipid metabolism in FHV RNA replication. The results presented in this report further support the well described crucial role that intracellular membranes play in positive-strand RNA virus replication [3-5], but emphasize that cellular lipids are key membrane constituents for this particular host-pathogen interaction. Furthermore, this report provides new details on the impact of specific lipid metabolism pathways on viral RNA replication, and in particular PC biosynthesis. The identification of specific lipid metabolism pathways is an essential first step in the rationale design of antiviral strategies that target cellular rather than viral components. Indeed, the recognition that cholesterol metabolism is important for hepatitis C virus replication in cultured cells [48,49] has led to direct clinical trials using cholesterol synthesis inhibitors [50].

The observation that PC is important for FHV RNA replication in cells is consistent with results published almost twenty years ago, which demonstrated that phospholipids enhance FHV RNA replication complex activity in isolated membrane fractions analyzed in vitro [21]. It also supports the hypothesis that one potential role cellular membranes play in viral RNA replication is to provide functional co-factors such as phospholipids for optimal RNA polymerase activity, and is consistent with published observations on the functional impact that phospholipids have on Semliki Forest virus nsP1 methyltransferase activity [51]. The precise mechanism(s) whereby phospholipids enhance FHV RNA replication complex activity is unknown, and there are multiple steps during process of viral RNA replication that could be influenced by these cellular components (see Fig. 1C). Interestingly, the observation that individual knockdown of *Cct1 *or *Cct2 *expression had an apparent preferential effect on subgenomic RNA3 production (Fig. 5) suggests that this particular step may be especially sensitive to cellular phospholipids. In addition to their potential roles as functional cofactors, membrane-resident lipids may also play other roles during RNA replication, such as providing a scaffold for replication complex targeting and assembly. Indeed, we recently demonstrated that FHV protein A is a lipid-binding protein with particular affinity for anionic phospholipids, including the mitochondrial-specific phospholipid cardiolipin [14]. Interestingly, we could not detect a significant physical interaction between FHV protein A and PC using in vitro assays [14], suggesting that PC may influence protein A activity indirectly or interact via some as yet unidentified intermediate protein or lipid.

Transcriptional array results suggested that FHV RNA replication stimulated PC synthesis in part via *Cct1 *or *Cct2 *upregulation, but the molecular mechanisms whereby FHV modulates lipid biosynthesis are unknown. One hypothesis is that FHV simply takes advantage of the cellular stress response to virus infection that may induce changes in phospholipid metabolism. However, published microarray results with cultured S2 cells infected with various pathogens, including viruses, bacteria, parasites, or fungi do not demonstrate a consistent upregulation of glycerophospholipid metabolism-related genes [44,47,52]. An alternative hypothesis is that FHV directly and specifically modulates glycerophospholipid metabolism. Interestingly, the enzyme encoded by *Drosophila Cct1 *is activated by cellular lipids, and in particular cardiolipin [24], suggesting a potential link with the observed protein A-cardiolipin interaction [14]. Phosphatidic acid, which represents the essential precursor in the synthesis of all glycerophospholipids [35] (see also Fig. 4B), also physically interacts with FHV protein A [14] and could serve as a potential conduit for regulation. Although these hypotheses are speculative and cannot directly explain transcriptional upregulation, they are readily testable with FHV using established in vitro and in vivo systems.

We focused on PC for this report, but our results do not exclude the potential important role of other cellular phospholipids in FHV RNA replication. Indeed, both transcriptomic (Fig. 2A) and functional genetic (Fig. 4A) analyses identified numerous additional candidate lipid metabolism-associated genes linked to FHV RNA replication. Furthermore, the lipidomics analysis examined total cellular membrane lipid content, whereas FHV RNA replication complexes localize to outer mitochondrial membranes and induce dramatic morphological and structural changes [11,15]. Although most glycerophospholipids, and in particular the highly abundant PC and PE, are widely distributed in membranes throughout the cell [29,53], detailed analyses of mitochondrial outer membrane lipids may reveal interesting and potentially more dramatic changes than we observed with the total cellular lipid analyses. Experiments are currently in progress to isolate and examine mitochondrial and submitochondrial fractions from cells with active FHV RNA replication complexes using well established techniques [54]. In addition, the observation that specific acyl chain modifications that result in longer unsaturated PC species were seen in FHV-infected cells suggests that particular lipid changes apart from the phospholipid head group also may play important roles in viral RNA replication. This is consistent with the demonstration that brome mosaic virus RNA replication is suppressed in yeast with a deletion of the enzyme Δ9 fatty acid desaturase [55]. FHV replicates robustly in *Saccharomyces cerevisiae *[12,56,57], and preliminary results suggest that FHV RNA replication in yeast is also influenced by alterations in PC metabolism (K. Stapleford and D. Miller, unpublished results). Phospholipid metabolism pathways and their regulation have been well studied in yeast [58], and therefore this genetically tractable host provides an excellent companion for parallel studies with *Drosophila *cells to further examine the impact of lipid metabolism on FHV RNA replication.

## Conclusions

In this study we demonstrate through a combination of complementary targeted and global analyses that glycerophospholipids, and in particular PC, play an important role in FHV RNA replication. These results enhance our understanding of the essential function that cellular membranes have in facilitating positive-strand RNA virus replication, and suggest that modulating cellular phospholipid synthesis may represent a novel approach for targeted antiviral development.

## Methods

### Cells and virus

*Drosophila *S2 cells were cultured in Schneider's *Drosophila *media (SDM) and transfected with inducible expression plasmids as previously described [17]. Sucrose gradient-purified FHV [11] was used for all infection experiments.

### Plasmids, antibodies, and chemicals

Standard molecular biology procedures were used for all cloning steps. The FHV RNA1 replicon expression plasmid pS2F1 (Fig. 1B), the protein A expression plasmid pS2FA-HA, and the control plasmid pS2LacZ have been previously described [17]. The control plasmid pS2F1_fs_, which contains an early frameshifting mutation in the protein A coding region (Fig. 1B) and hence will not produce a functional cis replicon transcript [6,12], was generated by cloning the *ScaI/BsrGI *fragment from pF1_fs _[6] into the *MscI/Acc65I *sites of pMT-V5/HisA (Invitrogen, Carlsbad, CA). Rabbit polyclonal antibodies against FHV protein A have been previously described [11], and antibodies against FHV protein B2 were generously provided by Paul Ahlquist (University of Wisconsin-Madison). Rabbit polyclonal antibodies against the hemagglutinin (HA) epitope tag or the voltage-dependent anion channel porin were purchased from Santa Cruz Biotechnology (Santa Cruz, CA) or Affinity Bioreagents (Golden, CO), respectively, while monoclonal antibodies against tubulin were purchased from the Developmental Studies Hybridoma Bank (University of Iowa, Iowa City, IA). All secondary reagents for ELISA and immunoblot analyses were purchased from Jackson Immunoresearch (West Grove, PA). The CTP:phosphocholine cytidyltransferase inhibitor miltefosine (1-hexadecylphosphorylcholine) was purchased from Calbiochem (San Diego, CA) and stored as a 100 mM stock solution in dimethylsulfoxide at -20°C. Oleic acid (*cis*-9-octadecenoic acid) was purchased from Sigma (St. Louis, MO) and stored as an undiluted stock solution at -20°C.

### FHV infection, replicon induction, and total RNA isolation

Cells were either infected with FHV at a multiplicity of infection of 10 as previously described [11] and harvested at 12 to 24 h after infection, or transiently transfected with pS2F1 and pS2LacZ as previously described [17] and harvested at 18 h after induction with 0.5 mM copper sulfate. For microarray and phospholipid analyses, S2 cells stably transfected with pS2F1 or pS2F1_fs _were induced with 1 mM copper sulfate and harvested at 18 h after induction. Total RNA was isolated using TRIzol reagent (Invitrogen) per the manufacturer's instruction. For microarray and RT-PCR analyses, RNA was further processed by digestion with RQ1 DNAse and subsequent purification with RNAsy columns (Qiagen, Valencia, CA). Total RNA purity, integrity, and concentration were checked by spectrophotometry and agarose gel electrophoresis, and samples were stored at -80°C until analysis.

### Affymetrix microarray data acquisition and analysis

Affymetrix *Drosophila *Genome 1.0 or 2.0 microarrays were used for FHV infection or replicon experiments, respectively, and biotinylated amplified cRNAs were produced using Affymetrix GeneChip target labelling kits per the manufacturer's instructions. Microarrays were scanned with an Affymetrix Scanner 3000, and complete original data files for all microarray experiments have been deposited in the Gene Expression Omnibus (GEO) database http://www.ncbi.nlm.nih.gov/geo/ under the accession number GSE15469.

Genomatix ChipInspector software package was used for primary microarray data analyses. This program uses a single probe method with an enhanced statistics package based on a SAM algorithm [59] that incorporates a *t*-test with a permuted artificial background to reduce false-positives. The following parameters were chosen to identify sets of up- or downregulated transcripts: (i) false-discovery rate of 1%; (ii) three probe minimum coverage; and (iii) 1.5-fold change from control. Similar results were obtained when microarray data were analyzed with the Affymetrix package of Bioconductor [60]. The FlyBase database (FB2008_09; http://flybase.org/) was used to identify Gene Ontology (GO) terms, genetic interaction partners, and yeast-human orthologs for *Drosophila *genes identified in the microarray analyses.

### Semiquantitative RT-PCR validation

First-strand cDNA synthesis was completed with equal amount of total RNA using oligo-dT primers and the Superscript II First Strand synthesis kit (Invitrogen) per the manufacturer's instructions. Ten-fold dilutions of cDNA were used for PCR reactions with gene-specific primer sets (Table 2) under the following conditions: 200 nM primers, 58°C annealing temperature, and 30 cycles. RT-PCR products were analyzed by agarose gel electrophoresis and ethidium bromide staining.

**Table 2:** Oligonucleotide sequences.

Name/description	Sequence (5' → 3')
Cct1-for	*GAGATGGCCGAGAAGTTGAG

Cct1-rev	*GGATTCCCTCGATTCTCACA

Cct2-for	*ACTTGGGCCTGAGACTGCTA

Cct2-rev	*CTTTCGGCATTCTGTCCATT

Act5C-for	ATGTGTGACGAAGAAGTTGCT

Act5C-rev	GTCCCAGTTGGTCACGATACC

FHV1-RNAi-for	***TAATACGACTCACTATAGGG***ATGACTCTAAAAGTTATTC

FHV1-RNAi-rev	***TAATACGACTCACTATAGGG***TCTGCTAGCGATAAAC

S2-RNAi-T7	***TAATACGACTCACTATAGGG***AGACCACGGGCGGGT

The T7 RNA polymerase promoter is in bold italics type for FHV1 RNAi-for, FHV1 RNAi-rev, and S2-RNAi-T7 oligonucleotides, and was also added to the 5' end of the Cct1 and Cct2 oligonucleotides (asterisks) that were used to generate dsRNAs.

### Phospholipid analysis

Total PC content was determined using a modified phospholipase D-based enzymatic method initially developed for measurement of plasma PC levels [27]. Briefly, S2 cells were pelleted at 1,000 × g for 5 min at 4°C, washed twice with Tris-buffered saline (TBS) (100 mM sodium chloride, 50 mM Tris, pH 7.2), lysed with 1% Triton X-100, and incubated with reaction buffer containing 50 mM Tris (pH 7.2), 0.64 M calcium chloride, 0.73 M *N*-ethyl-*N*-(2-hydroxy-3-sulfopropyl)-3,5-dimethoxyanaline, 0.73 M 4-aminoantipyrine, 120 U/ml PC-specific phospholipase D, 0.5 U/ml choline oxidase, and 20 U/ml peroxidase. Reactions were incubated at 37°C for 2 h and the absorbance at 595 nm was measured using a FLUOstar Omega microplate reader (BMG Labtech, Durham, NC). Quantitative PC concentrations were calculated using a standard curve generated with purified PC. Parallel total protein concentrations were determined with cell lysates prepared using TBS and 1% *n*-octyl-β-D-glucopuranoside and BioRad total protein reagent with bovine serum albumin as the standard. For analysis of individual cellular phospholipids, S2 cells were pelleted, washed twice with TBS, and lipids were extracted with chloroform:methanol using the method of Bligh and Dyer [61]. Final extract samples were dried under vacuum and analyzed for polar lipid content by ESI-MS/MS at the Kansas Lipidomics Research Center http://www.k-state.edu/lipid/lipidomics/index.htm.

### dsRNA production and RNAi induction

The O'Farrell RNAi library was purchased from the *Drosophila *Genome Research Center (DGRC). This collection contains 300-600 bp double-stranded cDNAs corresponding to greater than 7,000 individual phylogenetically conserved *Drosophila *genes [30], which can all be amplified by PCR using a common oligonucleotide primer (S2-RNAi-T7) that contains a GC-rich linker and T7 RNA polymerase promoter (Table 2). PCR products were generated using Eppendorf Mastermix with 200 nM primers, annealing temperature of 62°C, and 40 cycles. If no product was obtained after the first amplification, a second PCR was done using an aliquot of the first reaction as a template. PCR products were analyzed by non-denaturing gel electrophoresis and ethidium bromide staining and purified using a PCR cleanup kit (Promega) per the manufacturer's instruction. To generate the cDNA templates for *Cct1 *and *Cct2 *validation studies, candidate RNAi targets that differed from the O'Farrell library constructs were identified using the E-RNAi program [62]. To generate control FHV RNA1-specific dsRNA we targeted nucleotides 40-735, which include the initiator AUG codon. RT-PCR with gene-specific primer sets that incorporated a T7 RNA polymerase promoter (Table 2) were used to generate PCR templates for dsRNA generation. Control *LacZ *and *Hsp83 *cDNAs were produced as previously described [17].

RNAs were generated using a T7 Megascript kit (Ambion) per the manufacturer's instruction, purified by phenol-chloroform extraction and isopropanol precipitation, and resuspended in RNAse-free water. dsRNAs were generated by heating samples to 65°C for 30 min and cooling slowly to room temperature. Samples were analyzed by non-denaturing agarose gel electrophoresis and ethidium bromide staining to ensure the formation of properly sized dsRNA products, quantitated by spectrophotometry, and stored at -20°C until used for RNAi studies. RNAi was induced in cultured S2 cells as previously described [17]. Briefly, 10 μg dsRNA was added to 10^6 ^cells in 0.5 ml serum-free SDM, incubated at 25°C for 1 h, 0.5 ml SDM with 20% fetal bovine serum was added and cells were incubated for an additional 48-72 h prior to FHV infection or replicon transfection.

### FHV RNA replication analyses

To facilitate the analysis of FHV RNA replication in a medium throughput format, an FHV protein B2 capture ELISA was developed. FHV protein B2-specific polyclonal antibodies for detection were purified from rabbit antisera by *Staphylococcus aureus *protein A affinity chromatography and biotinylated with 6-(biotinamidocaproylamido)caproic acid *N*-hydroxysuccinimide ester (Sigma) per the manufacturer's instructions. For capture antibodies, total immunoglobulins from FHV protein B2 antisera were isolated by saturated ammonium sulfate precipitation. Flexible microassay plates were coated with 5 μg/ml capture antibody in phosphate-buffered saline (PBS) (100 mM sodium chloride, 50 mM sodium phosphate, pH 7.4) overnight at 4°C, blocked with 1% non-fat milk, and S2 cell lysates in PBS with 0.5% Triton X-100 were incubated in duplicate wells for 2 h at room temperature. Plates were washed extensively with TBS and 0.1% Tween 20, incubated with 1.25 μg/ml biotinylated detection antibody followed by streptavidin-alkaline phosphatase. Plates were developed with 1 mg/ml *p*-nitrophenyl phosphate in 50 mM sodium carbonate buffer (pH 10.0) with 1 mM magnesium chloride and the absorbance at 405 nm was measured with the microplate reader described above. Initial optimization experiments demonstrated a high sensitivity for this assay, where lysates from less than ten S2 cells expressing a pS2F1-based replicon produced a positive signal (data not shown). Northern blot analyses for FHV-specific RNAs, immunoblot analyses for protein A accumulation, saponin-mediated permeabilization and differential centrifugation, and β-galactosidase assays were done as previously described [17,22].

### Viability assays

Viability assays were performed using 3- [4,5-dimethylthizol-2-yl]-2,5-diphenyltetrazolium bromide (MTT) as previously described [63].

### Statistical analyses

Microarray statistical analyses are described above. For additional statistical analyses a two-tailed *t *test assuming equal variances was used and a *p *value of < 0.05 was considered significant. All results are representative or a composite of at least three independent experiments, where quantitative data represent the mean ± standard errors of the mean.

## Authors' contributions

KMC, KAS, and DJM conducted all the experiments. DJM wrote the manuscript and coordinated the research efforts. All authors read and approved the final manuscript.

## Supplementary Material

Additional file 1 **Complete list of *Drosophila *genes up- or downregulated in response to FHV infection**. *Drosophila *genes that showed significant up- or downregulation after FHV infection are listed in an Excel spreadsheet and include fold change, Flybase ID, gene symbol, gene name, and Gene Ontology (function, process, and compartment) terms curated from the Flybase database http://flybase.org/. Click here for file

Additional file 2 **Complete list of *Drosophila *genes upregulated in response to FHV replicon expression**. *Drosophila *genes that showed significant upregulation after FHV replicon expression are listed in an Excel spreadsheet as described above for Additional File 1. Click here for file

Additional file 3 **Complete list of *Drosophila *genes commonly upregulated after both FHV infection and replicon expression**. *Drosophila *genes that showed significant upregulation after FHV infected and replicon expression are listed in an Excel spreadsheet and include fold change, Flybase ID, CCG number, gene symbol, gene name, Gene Ontology (function, process, and compartment) terms, genetic interaction partners, and yeast and human orthologs, curated from the Flybase database http://flybase.org/. Click here for file

Additional file 4 **Phospholipid levels in *Drosophila *S2 cells infected with FHV treated with miltefosine or oleic acid**. Levels of individual lysoPC, PC, lysoPE, PE, PG, PI, PS, and PA species as determined by ESI-MS/MS are expressed as the molar percentage of total phospholipids content and listed in an Excel spreadsheet. Click here for file
